# Navigating the Challenges: Identifying Coping Strategies to Empower Direct Care Workers in Nursing Homes

**DOI:** 10.1093/geroni/igaf122.2141

**Published:** 2025-12-31

**Authors:** Alfred Boakye, Jennifer Morgan

**Affiliations:** University of Maryland, Baltimore, Baltimore, Maryland, United States; Georgia State University, Atlanta, Georgia, United States

## Abstract

A growing body of literature examines the challenges direct care workers face in the nursing home setting. Workforce shortages, low job quality, high workloads, and rising acuity make this context particularly fraught. Less attention has been devoted to exploring how nursing home staff can manage the everyday trauma they experience to improve their quality of life and work in the light of the multilayered social-based vulnerabilities inside and out of nursing homes. This study examines the various individual, organizational, and community coping strategies to help mitigate the challenges impacting direct care workers in nursing homes. Using a narrative exploratory design, we conducted 25 semi-structured interviews with certified nurse assistants recruited from two nursing home sites in the southeastern part of the United States. Member-checking or respondent validation was used to ensure accuracy and credibility. We find that individual strategies (e.g., faith/religion, personality traits/learned behaviors, recreational/self-care activities, and family history/experiences), organizational strategies (e.g., better compensation, developmental training & extracurricular/self-care activities), and community/family strategies (e.g., support groups, i.e., family & non-kin family) were instrumental in quality of life outcomes for certified nurse assistants as well as person-centered care for residents. Adopting and implementing coping strategies is foundational to building support for staff and ensuring they can understand and manage trauma and strengthen relationships. The study findings have implications for how to address multiple layers of factors (e.g., policy, organizational practices, and culture change initiatives) that impact carework and resident care in ways that support resilience-building and, ultimately, empowerment practices.

